# A quasi‐experimental study into the effects of naps and therapy glasses on fatigue and well‐being

**DOI:** 10.1111/jonm.13172

**Published:** 2020-10-22

**Authors:** Marianne van Woerkom

**Affiliations:** ^1^ Department of Human Resource Studies Tilburg University Tilburg The Netherlands; ^2^ Department of Psychology, Education and Child Studies Erasmus University Rotterdam Rotterdam The Netherlands

**Keywords:** fatigue, light therapy, napping, night shift work, nursing, psychological well‐being, quasi‐experiment

## Abstract

**Aim:**

To investigate the effects of a napping facility and therapy glasses on fatigue and well‐being at the end of the night shift.

**Background:**

Night shift work has adverse effects on fatigue and well‐being.

**Methods:**

A quasi‐experimental study was conducted, and data were collected on 243 night shifts of 95 nurses who had either access to a napping facility, therapy glasses, both facilities or no facilities. Multilevel analyses were conducted to predict fatigue and well‐being.

**Results:**

Night shifts of nurses having access to both facilities were associated with less fatigue and more well‐being. The use of therapy glasses related negatively to fatigue and positively to well‐being. The use of the napping facility was not associated with fatigue and well‐being. However, having slept while napping and sleeping time during napping were negatively associated with fatigue and positively associated with well‐being.

**Conclusion:**

Therapy glasses and sleeping in a napping facility can be effective interventions in reducing the adverse effects of night shift work.

**Implications for Nursing Management:**

Therapy glasses seem an effective investment to facilitate the well‐being of nurses. To enhance sleeping during napping, it is worthwhile to let nurses get accustomed to the napping facility and customize settings to personal preferences.

## 1 BACKGROUND

For many nurses, working in night shifts is a demanding characteristic of their job (Happell et al., 2013) that has been associated with increased levels of fatigue (Costa, 2010) and decreased levels of psychological well‐being (Ferri et al., 2016). This is a severe problem, since nurse fatigue and well‐being are directly related to the quality and safety of the care they give (Korompeli et al., 2013; Querstret et al., 2020). The negative consequences of shift work for health and well‐being are mainly due to the fundamental misalignment between the circadian rhythm of the endogenous biological clock and the timing of the sleep/wake cycle (James et al., 2017). The tendency to sleep is regulated by the interplay between a homeostatic pressure to sleep that increases with each hour of wakefulness and a circadian alerting signal that encourages wakefulness (Wickwire et al., 2017). Shift workers need to sleep when the circadian alerting signal is strongest, and must be alert and ready to work when circadian alerting signals are at their lowest, and the pressure to sleep is at its strongest. Because most nurses work in rotating shifts and return to normal hours on their days off, their circadian system never fully adapts, leading to sleep deficits, fatigue and a variety of physical and mental symptoms (Costa, 2010; Shields, 2002). Circadian misalignment also has adverse consequences for the psychological well‐being of shift workers due to the changes in the cortisol rhythm that activate the fear system and blunt the reward system, thereby leading to heightened stress activity and impaired emotion regulation (James et al., 2017). Moreover, working at night also limits contact with psychosocial factors that protect against stress, such as participation in leisure time and family activities (Shields, 2002).

One potential intervention to reduce fatigue and promote well‐being during the night shift is to provide nurses with a napping opportunity during the night shift (Dalky et al., 2018). This could potentially enhance the recovery from the ongoing pressure on personal capacities, reinforce the synchronization of the circadian rhythm and compensate for sleep deprivation (Davy & Göbel, 2013). However, results of napping interventions are still inconsistent (Li et al., 2019). Some studies verified that persons who took a nap during the night shift were less fatigued in the morning (Barthe et al., 2016; Slanger et al., 2016; Smith‐Coggins et al., 2006), that night shift napping compensates for the shorter sleep at home (Ribeiro‐Silva et al., 2006) or that naps facilitate the readjustment to diurnal life (Daurat & Foret, 2004). However, other studies did not confirm this finding (Chang et al., 2015; Neville et al., 2017; Oriyama et al., 2013). Inconsistencies may be due to the duration and timing of the nap, disturbing environmental elements such as noise and lighting (Barthe et al., 2016; Querstret et al., 2020; Wendsche et al., 2017), and the different industries in which a nap opportunity has been tested (Li et al., 2019). To reduce the impact of a suboptimal environment for napping, an innovative napping facility was tested with a privacy screen, a temperature regulator, a noise‐cancelling headset that could produce relaxing sounds and a massage system. Furthermore, whereas most (quasi) experimental studies on night shift napping test the effect of relatively long naps, averaging 59 min (Barthe et al., 2016), forty minutes (Smith‐Coggins et al., 2006) and thirty minutes (Chang et al., 2015), a relatively short twenty‐minute nap was tested that may be more feasible in a health care setting, especially in eight‐hour shifts. Because it is still unclear to what extent the effect of only resting during the napping time is equally effective as actually sleeping (Barthe et al., 2016), the effect of actually sleeping during the napping time was also investigated.

Another potential intervention to alleviate the adverse effect of night shift work is the exposure to bright light, which is the dominant environmental time cue that entrains the human circadian clock to a 24‐hr day and determines whether the internal clock is phase‐delayed or phase‐advanced (Huang et al., 2013; Querstret et al., 2020). Light can induce both circadian and acute physiological responses in humans (Cajochen et al., 2000), and research suggests that exposure to bright light during night‐time hours reduces the release of melatonin, which is associated with lower level of sleepiness and higher levels of alertness and night‐time performance (Burgess et al., 2002). Some studies have found favourable effects of bright light during night work on stress and burnout symptoms among nurses (Kakooei et al., 2009), perceived sleep effectiveness (Jensen et al., 2016) and total sleep time (Boivin et al., 2012; Rahman et al., 2013). However, a recent systematic review of studies examining the effectiveness of light during night shift work concluded that the evidence supporting a positive effect of controlled light exposure is still too weak to draw definite conclusions (Slanger et al., 2016). Furthermore, previous studies have mostly investigated static, location‐bound lighting solutions, which may be difficult to implement in the extremely dynamic working environment of nurses and cannot be adjusted to personal needs (Aarts et al., 2020). For this reason, the effect of light therapy glasses on fatigue and psychological well‐being was tested at the end of the night shift.

The aim of this study was to study the effectiveness of an innovative napping facility and an innovative light therapy intervention on fatigue and psychological well‐being at the end of the night shift. Furthermore, it was also investigated to what extent nurses were actually able to sleep during their napping time and how that was related to fatigue and well‐being. Many studies on interventions that aim to reduce the adverse effects of night shift work take place in a laboratory setting that simulate shift work (Signal et al., 2009; Slanger et al., 2016). This makes it difficult to generalize the effects of these studies to occupational settings in which an optimal implementation of these interventions, or the actual use of the facilities that are offered may not always be feasible. This study took place in a naturalistic setting of night shifts of nurses in a hospital, thereby ensuring ecological validity.

Furthermore, there is scant knowledge on the effect of night shift interventions on psychological well‐being. However, since happy workers perform better (Lyubomirsky et al., 2005), are more helpful and display more organisational citizenship behaviour (Kaplan et al., 2009), and are more creative and innovative (Amabile et al., 2005), psychological well‐being seems an important state for nurses who need to work in an increasingly dynamic and demanding work environment (Verhaeghe et al., 2006).

## 2 METHODS

A quasi‐experimental study was conducted with a pre–post design repeated over 3 night shifts.

### 2.1 Participants and procedure

In a first step, ward managers in a Dutch hospital were informed about the study and requested to participate. Five wards (intensive care, paediatric care, emergency, medium care and orthopaedic) were willing to participate. Participants were nurses working rotating night shifts (from 10.30 p.m. to 7.30 a.m.). This means that this study is focused on eight‐hour shifts as compared to other countries such as the United States where the norm is 12‐hr shifts. Cluster randomization (Nielsen & Miraglia, 2017) was applied by randomly assigning wards to different condition interventions. Nurses working at the intensive care were provided with a napping facility, nurses working at the paediatric care were provided with light therapy glasses, and nurses working at the emergency ward were provided with both a napping facility and light therapy glasses. Nurses who worked at the medium care ward or the orthopaedic ward were not provided with any of the facilities. A kick‐off event was organised in order to inform the nurses about the study and the interventions. The purpose and content of the study were presented during this event; however, the anticipated effects on the study variables were not addressed. Nurses of all five wards were asked to fill in a paper‐and‐pencil survey booklet including questions regarding their levels of fatigue and psychological well‐being at the start of their night shift (T1) and at the end of their night shift (T2) for a period of three nights. Furthermore, a one‐time general questionnaire was included to collect data regarding age, gender, full‐time/part‐time contract, educational level, tenure as a nurse, tenure working in nightshifts and average number of night shifts per month. As soon as the nurses had completed the surveys on three nights (including a before and after night shift measurement), they were requested to hand in their survey booklet. Since the participants had different types of contracts (full‐time vs. part‐time), and different schedules with varying numbers of night shifts per month, a data collection period of 2 months was chosen to ensure that sufficient participants would be able to fill in the questionnaire on three night shifts.

The five participating departments had on average 30 nurses who work at least three nights shifts in a period of 2 months. A calculation in G*power (Faul et al., 2009) indicated that when 75 participants (50 per cent response rate) would respond on at least two nights (300 nights), this would give a sufficient sample size to detect small to medium effect sizes (f2 = .05) in a regression model with eight predictors.

### 2.2 The interventions

#### 2.2.1 The napping facility

The nurses from the emergency ward and the intensive care ward were given the opportunity to take a 20‐min nap whenever it was most convenient to them in an innovative nap facility that was placed in a separate room. The nap facility was commercially available (Ahrend Loungescape Powernap, Royal Ahrend N.V.). A duration of 20 min fitted best into employee work schedules and allowed for approximately 10 min of sleep, which has been proven to be an effective sleeping time that prevents sleep inertia (Brooks & Lack, 2006). The bed was equipped with a privacy screen, a temperature regulator and a noise‐cancelling headset that could produce relaxing sounds. Because massage has been associated with indicators of relaxation such as reduced levels of anxiety, depression, blood pressure, heart rate and cortisol level (Moyer, 2004), the mattress contained a whole‐body vibration massage system. The strength of the vibrations targeting individual body parts could be adjusted with an app. After 20 min, the massage system would stop. Participants were free to use or not use any of the features. Participants had to set the alarm on their mobile phone to wake them. Due to budget restrictions, only one nap facility was available per ward.

#### 2.2.2 Light therapy glasses

The nurses from the paediatric care and the emergency ward were provided with light therapy glasses (Propeaq, 2019) with integrated LEDs in the frame that could expose the participant to blue light. The blue light had a wavelength of 468 nanometre and an intensity of 35 lux. A recent study (Aarts et al., 2020) indicated that the use of these light therapy glasses reduced sleepiness of nurses only during the first night shift in a row of three night shifts. In accordance with the guidelines of the manufacturer (Propeaq, 2019), nurses were instructed to wear the glasses for 30 min, between 2 a.m. and 4 a.m., right before they experienced a lack of energy. Participants who could also make use of the nap facility were instructed not to wear the glasses during or right before their napping time. Nurses were able to work while wearing the blue light therapy glasses.

### 2.3 Measures

#### 2.3.1 Use of facility

It was measured whether nurses made use of the facilities that were provided by them with the items ‘Did you make use of the [napping facility/ blue light glasses] (0 = no, 1 = yes)’. Furthermore, participants were asked whether they slept during the use of the napping facility (0 = no, 1 = yes), and if so, for how many minutes. They were also asked for how many minutes the glasses were worn and whether the glasses were worn between 2 a.m. and 4 a.m.

#### 2.3.2 Fatigue

Fatigue was measured by using the Dutch version of the 10‐item Fatigue Assessment Scale (FAS) (Michielsen et al., 2003). The FAS includes ten statements about how individuals 'usually' feel. Since the present study focused on momentary fatigue, the items were adapted to refer to how respondents felt at the start of their night shift (T1) and at the end of their night shift (T2), whereas the answering scale was changed to a 5‐point Likert scale ranging from 1 (‘strongly disagree’) to 5 (‘strongly agree’). Example items are 'I am bothered by fatigue', 'Physically, I feel exhausted', 'I have problems with thinking clearly' and 'I can concentrate well'. The Cronbach's *α* was .92 (T1) and .92 (T2). Intraclass correlations (ICC1; Bliese, 2000), indicating the proportion of the total amount of variance accounted for by the individual level (instead of the night level), were .39 for fatigue T1 and .51 for fatigue T2.

#### 2.3.3 Psychological well‐being

Psychological well‐being was measured with a Dutch version of the Everyday Feeling Questionnaire (EFQ), which comprised 10 items (Uher & Goodman, 2010) ranging from 1 (‘strongly disagree’) to 5 (strongly agree). The EFQ measures psychological state‐like elements such as optimism (‘At this moment, I am positive about the future’), enjoyment (‘At this moment, I am able to enjoy life), self‐esteem (‘At this moment, I am positive about myself’) and calmness (‘At this moment, I am stressed’ (R). The items were slightly adapted by adding ‘at the moment’ referring to the momentary level. The Cronbach's *α* for T1 was *a* = .847, and T2, *a* = .850. Intraclass correlations were .47 for psychological well‐being T1 and .59 for psychological well‐being T2.

### 2.4 Analyses

An analysis of missing data indicated that there was no missing data on the study variables, except for one value on the control variable consecutive night shift. Therefore, listwise deletion was used in all analyses. To investigate pre‐intervention differences between wards, one‐way ANOVAs were conducted, and to investigate the relationships between all the study variables, Pearson's correlations were computed. Since the observations referred to 243 nights that were nested in 95 respondents, multilevel hierarchical regressions were conducted using the linear mixed‐effects model procedure in SPSS (Heck et al., 2010) such that the effects of the night‐level variables were examined while accounting for the non‐interdependence of observations within individuals (Diez‐Roux, 2000). All analyses, were controlled for baseline levels of the dependent variables (fatigue and psychological well‐being), measured at the beginning of the night shift. Deviance scores (differences in the −2 log likelihood) Were computed to compare the different models with a baseline model, including only consecutive night shift, the level of the dependent variable at the beginning of the night shift and the between‐individuals level as only predictors, and to test their significance (Bickel, 2007). Measures of model fit for all models were then obtained by comparing deviance scores using a chi‐squared distribution table (Bryk & Raudenbush, 1992).

## 3 RESULTS

### 3.1 Sample characteristics

In total, the sample comprised data that were collected in 243 night shifts nested in 95 participants of which 15 were males (15.8%), and 78 were females (82.1%) (two persons did not indicate their gender). The average age of the participants was 36.168 (*SD* = 10.308). Twenty‐nine nurses (30.5%) worked at a emergency ward, 12 nurses (12.6%) worked at an orthopaedic ward, 18 nurses (18.9%) worked at an intensive care ward, 16 nurses (16.8%) worked at a medium care ward, and 20 nurses (21.1%) worked at a paediatric ward Table 1 presents an overview of the participating wards, interventions and the N of participants and nights. A one‐way ANOVA indicated no significant differences between different wards on the levels of fatigue at the beginning of the night shift (*F*(4, 242) = 1.021, *p* = .397). However, for psychological well‐being T1, there were significant differences (*F*(4, 242) = 7.513, *p* = .000) between the averages of psychological well‐being at the orthopaedic ward (3.48) compared with those at the emergency ward (3.92, *p* = .000), IC ward (3.86, *p* = .007) and medium care ward (4.06, *p* = .000), and between the paediatric ward (3.70) and the medium care ward (4.06, *p* = .010). However, since in all analyses we controlled for the baseline scores of the dependent variables, this was not considered this as problematic for the results.

**TABLE 1:** Wards, interventions and *N* participants/nights

Ward	Intervention	*N* participants/nights
Intensive care	Napping facility	18/45
Paediatric care	Light therapy glasses	20/54
Emergency	Napping facility and light therapy glasses	29/74
Medium care	No intervention	16/37
Orthopaedic	No intervention	12/33

The large majority of the respondents were licensed practical nurses (33.7 per cent) or registered nurses with a BSN degree (57.9 per cent). Participants worked on average five night shifts per month (*SD* = 1.87), and 29.2 per cent of them had a part‐time contract. Nurses who had access to the nap facility made use of this provision in **77**% of the nights (92 nights). In 39.5% of these occasions, nurses actually fell asleep. Even though participants were instructed to use the napping facility for a maximum of 20 min, the estimated time being asleep ranged from 2 min to 30 min, with an average of 5.69 min (*SD* = 8.647). Nurses who had light therapy glasses at their disposal wore these glasses in 66.6% of all the nights (84 nights) for on average 29.88 min (*SD* = 5.03). In 96.4% of the nights, the glasses were worn for the instructed 30 min.

### 3.2 Means, standard deviations and correlations

Table 2 reports the means, standard deviations and correlations between the study variables. Please note that the means and standard deviations of the variables use napping facility, did you sleep?, time asleep, use therapy glasses, and time using glasses are based on the whole sample (with participants not having access to the facilities coded to 0) and not just the subsample who had access to these facilities. As can be seen, the use of the nap facility was not related to fatigue T2 (*r* = −.08, *ns*) or psychological well‐being T2 (*r* = −.09, *ns*). However, having slept in the nap facility and time being asleep were negatively related to fatigue T2 (respectively *r* = −.19, *p <* .01; and *r* = .28, *p <* .01) and positively related to psychological well‐being T2 (respectively *r* = −.17, *p <* .01; and *r* = .20, *p <* .01). Also, the use of the blue light therapy glasses was negatively related to fatigue T2 (respectively *r* = −.16, *p <* .01) and positively related to psychological well‐being T2 (respectively *r* = .14, *p <* .01).

**TABLE 2:** Means, standard deviations and correlations between the study variables

	Mean	*SD*	1.	2.	3.	4.	5.	6.	7.	8.	9.	10.	11.
1. Gender^a^	1.83	.38											
2. Age	36.31	10.28	−.08										
3. Cons. night shift^b^	2.05	.96	−.02	.09									
4. Use napping facility^c^	.38	.49	−.15*	.29**	.05								
5. Did you sleep?^c^	.20	.40	.00	.12	.04	.61**							
6. Time asleep ^d^	2.79	6.68	.04	.06	.06	.54**	.84**						
7. Use therapy glasses^c^	.49	.50	−.04	.09	.05	−.10	.01	.04					
8. Time using glasses^e^	10.33	14.54	.16*	.05	.02	−.14*	.03	.07	.73**				
9. Fatigue T1	2.47	.75	.06	−.09	.02	−.03	−.03	−.03	.00	.01			
10. Fatigue T2	3.19	.79	.15*	−.19**	−.16*	−.08	−.19**	−.28**	−.16*	−.11	.48**		
11. Ps. well‐being T1^f^	3.82	.54	.04	.014	−.03	.07	.12	.09	−.04	−.03	−.57**	−.33**	
12. Ps. well‐being T2^f^	3.55	.55	−.08	.014	.07	.09	.17**	.20**	.14*	.06	−.31**	−.62**	.60**

Note

*N* = 243 nights, 95 participants.

^a^ 1 = male, 2 = female.

^b^ Consecutive night shift.

^c^ 0 = no, 1 = yes.

^d^ Ranging from 0 to 30 min.

^e^ Ranging from 0 to 60 min.

^f^ Psychological well‐being.

* *p* < .05,

** *p* < .01.

### 3.3 The effects of the experimental conditions

Table 3 reports the results of multilevel regression analyses that predicted fatigue T2 and psychological well‐being T2 from the facilities that were made available to the different wards. As can be seen from this table, being provided with both a nap facility and light therapy glasses had a negative effect on fatigue T2 and a positive effect on psychological well‐being T2 (respectively *B* = −.42, *p* < .01; and *B* = .31, *p* < .01).

**TABLE 3:** Multilevel Regression Analyses Predicting Psychological Well‐being T2 and Fatigue T2 from the facilities that were provided

Variable	Fatigue T2^d^			Psych. Well‐being T2^e^		
	Estimate	*SE*	*p*	Estimate	*SE*	*p*
Constant	2.56***	.19	.00	1.43***	.22	.00
Consecutive night shift	−.10*	.04	.01	.03	.03	.31
Fatigue T1	.41***	.05	.00			
Psych. Well‐being T1				.50***	.05	.00
Nap facility and th. glasses^a^	−.42**	.14	.00	.31**	.09	.00
Nap facility^b^	−.02	.16	.91	.05	.10	.62
Therapy glasses^c^	−.11	.15	.46	.08	.09	.38
Between‐individuals level						
Variance random intercept	.30***	.04	.00	.05**	.02	.01
−2log likelihood	461.57			256.96		
Deviance change (∆*χ* ^2^)^a^	10.41*			12.91**		
AIC	477.57			272.96		
BIC	505.42			300.80		

Note

*N* = 243 nights, 95 participants. Reported values for each model are estimates of the effect, comparable to unstandardized regression coefficients in standard multiple regression. Results of analyses with the control variables age and gender were not substantially different.

^a^ Emergency aid ward = 1, else = 0.

^b^ IC ward = 1, else = 0.

^c^ Paediatric ward = 1, else = 0.

^d^ To calculate the deviance change, the model was compared with a model 0, with consecutive night shift, baseline level of fatigue and the between‐individuals level as only predictors (*df* = 3).

^e^ To calculate the deviance change, the model was compared with a model 0, with consecutive night shift, baseline level of psychological well‐being and the between‐individuals level as only predictors (*df* = 3). Comparison between model 0 and model 1: *df* = 2; comparison between model 0 and model 2: *df* = 2.

* *p* < .05,

** *p* < .01,

*** *p* < .001.

### 3.4 The effects of the use of the facilities

Table 4 reports the results of multilevel regression analyses that predicted fatigue T2 from the use of the napping facility and the light therapy glasses (model 1), from having slept in the napping facility and the use of the therapy glasses (model 2) and from the time slept and the use of the therapy glasses (model 3). As can be seen in model 1, making use of the napping facility was not associated with fatigue T2 (*B* = −.12, *ns*). However, having slept in the napping facility (model 2) and particularly the time that one was asleep (model 3) were associated with lower levels of fatigue (T2) (respectively *B* = −.29, *p* < .01; and *B* = −.03, *p* < .001). Furthermore, making use of light therapy glasses was in all models negatively associated with fatigue T2 (respectively *B* = −.24, *p* < .05; *B* = −.23, *p* < .05; and *B* = −.23, *p* < .05).

**TABLE 4:** Multilevel Regression Analyses Predicting Fatigue T2 from Use of the Facilities

Variable	1			2			3		
	Estimate	*SE*	*p*	Estimate	*SE*	*p*	Estimate	*SE*	*p*
Constant	2.60***	.18	.00	2.60***	.18	.00	2.60***	.17	.00
Consecutive night shift	−.12**	.04	.00	−.11**	.04	.01	−.11**	.04	.01
Fatigue T1	.41***	.06	.00	.41***	.06	.00	.41	.06	.00
Use napping facility^a^	−.12	.10	.22						
Did you sleep?^a^				−.29**	.11	.00			
Time asleep^b^							−.03***	.01	.00
Use glasses^a^	−.24*	.11	.02	−.23*	.10	.03	−.23*	.10	.03
Between‐individuals level									
Variance random intercept	.15**	.04	.00	.14**	.04	.00	.13**	.04	.00
−2log likelihood	466.16			460.70			450.81		
Deviance change (∆*χ* ^2^)^a^	5.82			11.28**			21.17***		
AIC	480.16			474.70			464.81		
BIC	504.53			499.06			489.18		

Note

*N* = 243 nights, 95 participants. Reported values for each model are estimates of the effect, comparable to unstandardized regression coefficients in standard multiple regression. Results of analyses with the control variables age and gender were not substantially different.

^a^ 0 = no, 1 = yes.

^b^ Ranging from 0 to 30 min. To calculate the deviance change, the models were compared with a model 0, with consecutive night shift, baseline level of fatigue and the between‐individuals level as only predictors (*df* = 3). Comparison between model 0 and model 1: *df* = 2; comparison between model 0 and model 2: *df* = 2; and comparison between model 0 and model 3: *df* = 2.

* *p* < .05,

** *p* < .01,

*** *p* < .001.

Table 5 reports the results of multilevel regression analyses that predicted psychological well‐being T2 from the use of the nap facility and the light therapy glasses (model 1), from having slept in the napping facility and the use of the therapy glasses (model 2) and from the time slept and the use of the therapy glasses (model 3). As can be seen in this table, making use of the napping facility (model 1) and having slept in the napping facility (model 2) were not associated with higher levels of psychological well‐being T2 (respectively *B* = .04, *ns*; and *B* = .11, *ns*). However, time slept (model 3) was positively related to psychological well‐being T2 (*B* = .01, *p < *.05). Furthermore, making use of light therapy glasses was in all models positively associated with psychological well‐being T2 (respectively *B* = .17, *p* < .05; *B* = .17, *p* < .05; and *B* = .16, *p* < .05).

**TABLE 5:** Multilevel regression analyses predicting psychological well‐being T2 from use of the facilities

Variable	1			2			3		
	Estimate	*SE*	*p*	Estimate	*SE*	*p*	Estimate	*SE*	*p*
Constant	1.37***	.23	.00	1.40	.22	.00	1.40***	.22	.00
Consecutive night shift	.04	.03	.14	.04	.03	.16	.04	.03	.19
Psychological well‐being T1	.52***	.05	.00	.51***	.06	.00	.51***	.05	.00
Use nap facility	.04	.06	.49						
Did you sleep?				.11	.07	.15			
Time asleep							.01*	.00	.03
Use therapy glasses	.17*	.07	.01	.17*	.07	.01	.16*	.06	.02
Between‐individuals level									
Variance random intercept	.06	.02	.00	.05**	.02	.01	.05**	.02	.01
−2log likelihood	263.67**			262.09			259.548		
Deviance change (∆*χ* ^2^)^a^	6.2*			7.79*			10.322**		
AIC	277.67			276.09			273.55		
BIC	302.04			300.45			297.91		

Note

*N* = 243 nights, 95 participants. Reported values for each model are estimates of the effect, comparable to unstandardized regression coefficients in standard multiple regression. Results of analyses with the control variables age and gender were not substantially different.

^a^ 0 = no, 1 = yes.

^b^ Ranging from 0 to 30 min. To calculate the deviance change, the models are compared with a model 0, with consecutive night shift, baseline level of psychological well‐being and the between‐individuals level as only predictors (*df* = 3). Comparison between model 0 and model 1: *df* = 2; comparison between model 0 and model 2: *df* = 2; and comparison between model 0 and model 3: *df* = 2.

* *p* < .05,

** *p* < .01,

*** *p* < .0.

## 4 DISCUSSION

Nurses who work on night shifts are at risk from the negative effects of stress‐related problems, with high rates suffering from fatigue (Thompson et al., 2017) and low levels of psychological well‐being (Ferreira et al., 2017). This is a serious problem, since nurse fatigue and well‐being have a direct impact on the quality and safety of the care they give (Korompeli et al., 2013).

The results of this study indicate that having access to both a napping facility and light therapy glasses reduces fatigue and boosts psychological well‐being at the end of the night shift. Nurses who were provided with only a napping facility or only light therapy glasses did not deviate from other nurses in terms of their fatigue and psychological well‐being at the end of the night shift. When looking at the actual use of the napping facility, the results suggest that using this facility without falling asleep does not reduce fatigue or boost psychological well‐being. This is in contrast to previous researchers who have suggested that rests may have a similar recuperating value to that of naps (Davy & Göbel, 2013). However, the results are in line with a study by Barthe et al. (2016) who found an effect of sleeping but not of resting on sleepiness. This study underlines the importance for future studies to differentiate between napping and sleeping, and to further investigate the conditions for falling asleep when using a napping facility.

The studies that have explored the impact of naps in health care settings vary strongly in the timing and the length of the naps that have been investigated (Querstret et al., 2020). Even though the design of the present study does not allow for causal relationships, it indicates that even very short naps with an average sleeping time of 5.69 min may be able to reduce fatigue and boost well‐being at the end of the night shift. Although the time asleep played an important role in the effectiveness of the nap, the maximum sleeping time (30 min) was still very limited. This is an interesting finding, since most field studies test the effect of longer nap times, varying between 40 min (Signal et al., 2009; Smith‐Coggins et al., 2006) and 3 hr (Palermo et al., 2015). However, long breaks can be highly impractical in health care settings where staff shortages and concerns about patient safety exist (Querstret et al., 2020) and do usually not fit in the work schedules of nurses, particularly when they work in eight‐hour shifts. Furthermore, whereas some studies (Smith et al., 2007; Smith‐Coggins et al., 2006) investigated the effects of naps within a specific time frame (e.g. between 2 a.m. and 3 a.m.) this is challenging in a naturalistic setting and dependent on factors such as availability of staff, patient acuity, team dynamics and the availability of nap facilities. The present study shows that self‐selected nap times can be an effective intervention. However, more research is needed to investigate the optimal timing of self‐selected naps, since naps taken closer to early morning hours may interfere with post‐shift sleep ability and may be less effective to mitigate sleepiness (Fallis et al., 2011).

A consistent effect was found of wearing light therapy glasses on fatigue and well‐being at the end of the night shift. Although this is in line with some studies (Leppämäki et al., 2003; Smolders & de Kort, 2014), a systematic review concluded that the evidence supporting a positive effect of controlled light exposure is still too weak to draw definite conclusions (Slanger et al., 2016). However, previous studies have mostly investigated static, location‐bound lighting solution that may not be suitable for hospital nurses who work in extremely dynamic working environments. One very recent study among 23 nurses (Aarts et al., 2020) indicated that the use of light therapy glasses reduced sleepiness of nurses only during the first night shift in a row of three nightshifts. The present study indicates that wearing light therapy glasses is also associated with higher levels of psychological well‐being at the end of the night shift and is independent of the consecutive night shift. Overall, the findings of this study may contribute to the discussion on effective countermeasures to relieve the negative effects of night shift work on nurses.

### 4.1 Strengths, limitations and future research

A strength of this study is that it is among the first to investigate the effect of innovative devices (i.e. a napping device with many technological features and light therapy glasses) in the naturalistic context of nursing. However, this study is also subject to four main limitations.

A first limitation concerns the allocation of nurses to the different conditions. Unfortunately, it was not possible to randomly allocate participants to experimental conditions. Instead, cluster randomization (Nielsen & Miraglia, 2017) we applied by assigning wards to different conditions. Since we controlled in all analyses were controlled for the baseline levels of the dependent variables, it is unlikely that differences in effects between the groups are attributable to individual differences at baseline. Furthermore, this study focused not only on the effect of the provision of both facilities but also interested on the effect of the actual use of the facilities. However, this design does not allow for causal attributions, and future studies should try to randomly allocate interventions to individual participants. Also, the sample size of 243 observations in a model with 8 predictors gave the analyses a power of .80 for small to medium effect sizes (f2 = .07), but only a power of .28 for small effect sizes (f2 = .02). Hence, it is possible that there were in fact small effects of the experimental conditions or the use of the facilities that this study could not detect.

Second, this study is mainly based on subjective perceptions of participants and does not include physiological or objective measures. This is particularly problematic in the case of the self‐reports of time slept, since subjects' reports of sleep are only moderately correlated with objectively measured sleep (Lauderdale et al., 2008). Future studies should therefore try to measure whether participants had slept and how long with more objective measures. Furthermore, existing scales for fatigue and psychological well‐being had to be adapted to the momentary level. Future studies should investigate the validity of these adapted scales.

Third, unfortunately, no information was available regarding the timing of the naps. Since a qualitative study indicates that naps earlier in the night have greater physiological benefits compared with naps later in the night (Fallis et al., 2011), future research should take the timing of the nap into account.

Fourth, although the facilities that were offered were not always used by nurses, no systematic data was collected regarding the reasons for this. Some participants indicated that some nights were too busy to make use of the nap facility and that the light therapy glasses were sometimes not used in order not to scare of patients, or because spectacle wearers felt they were not comfortable. Future research should therefore include variables regarding the reasons for not making use of the facilities. Furthermore, even though participants were given some time during the kick‐off meeting to familiarize themselves with the facilities, this may have been insufficient. Possibly, participants were hesitant to make use of the nap facility because they were afraid of sleep inertia. Another avenue for future research is therefore to allow participants more time to get used to the facilities, and then evaluate the outcomes.

### 4.2 Practical implications

Light therapy glasses and innovative napping facilities can be effective interventions in reducing the adverse effects of night shift work. Light therapy glasses are relatively cheap (approximately 200 euro) and can be shared among nurses. Because these glasses showed consistent effects on fatigue and psychological well‐being at the end of the nightshift and can be implemented without disrupting the work routine, they seem to be a worthwhile investment for hospitals. The innovative napping facility is more expensive (approximately 7,000 euro) and more complicated to implement. Because this study indicated that a napping facility is only effective when it helps people to actually sleep, it is probably worthwhile to invest time in letting nurses become accustomed to this facility and experiment with customizing the settings regarding sound, temperature and massage to their personal preferences.

## ETHICAL APPROVAL

Ethical Review Board of Tilburg University (reference EC‐2019.83).
